# Oridonin inhibits the migration and epithelial‐to‐mesenchymal transition of small cell lung cancer cells by suppressing FAK‐ERK1/2 signalling pathway

**DOI:** 10.1111/jcmm.15106

**Published:** 2020-03-13

**Authors:** Linhao Xu, Yanli Bi, Yizhou Xu, Zhuocheng Zhang, Wenjie Xu, Sisi Zhang, Jian Chen

**Affiliations:** ^1^ Department of Cardiology Affiliated Hangzhou First People’s Hospital Zhejiang University School of Medicine Hangzhou China; ^2^ School of Basic Medical Sciences & Forensic Medicine Hangzhou Medical College Hangzhou China; ^3^ Translational Medicine Research Center Affiliated Hangzhou First People’s Hospital Zhejiang University School of Medicine Hangzhou China; ^4^ Department of Clinical Laboratorial Examination Air Force Hangzhou Special Service Recuperation Center Sanatorium Area 3 Hangzhou China

**Keywords:** focal adhesion kinase, migration, oridonin, small cell lung cancer

## Abstract

Small cell lung cancer (SCLC) is a severe malignant with high morbidity; however, few effective and secure therapeutic strategy is used in current clinical practice. Oridonin is a small molecule from the traditional Chinese herb *Rabdosia rubescens.* This study mainly aimed to investigate the role of oridonin on inhibiting the process of H1688, a kind of small cell lung cancer cells from human. Oridonin could suppress H1688 cell proliferation and induce their apoptosis in a high dosage treatment (20 μmol/L). Meanwhile, cell migration was suppressed by oridonin (5 and 10 μmol/L) that did not affect cell proliferation and apoptosis. The expression level of E‐cadherin was significantly increased, and the expression of vimentin, snail and slug was reduced after administration of oridonin. These expression changes were associated with the suppressed integrin β1, phosphorylation of focal adhesion kinase (FAK) and ERK1/2. In addition, oridonin (5 and 10 mg/kg) inhibited tumour growth in a nude mouse model; however, HE staining revealed a certain degree of cytotoxicity in hepatic tissue after treatment oridonin (10 mg/kg). Furthermore, the concentration of alanine aminotransferase (ALP) was significantly increased and lactate dehydrogenase (LDH) was reduced after oridonin treatment (10 mg/kg). Immunohistochemical analysis further revealed that oridonin increased E‐cadherin expression and reduced vimentin and phospho‐FAK levels in vivo. These findings indicated that oridonin can inhibit the migration and epithelial‐to‐mesenchymal transition (EMT) of SCLC cells by suppressing the FAK‐ERK1/2 signalling pathway. Thus, oridonin may be a new drug candidate to offer an effect of anti‐SCLC with relative safety.

## 1 INTRODUCTION

According to a previous study, lung cancer has a highest cancer death rate among different cancer diseases.^1^ There were two different histological subtypes in lung cancer: small cell lung cancer (SCLC) and non‐small cell lung cancer (NSCLC). Although only approximately 10%‑15% of lung cancers is SCLC, SCLC is the most aggressive and rapidly growing lung subtype.^2^ According to the previous study, the 5‐year survival rate of SCLC is <5%, as most SCLC patients are diagnosed at a late period.^1^ In the clinic, cisplatin or carboplatin plus etoposide is currently commonly used; however, this drug is not used widely in clinical because of its lower efficacy and highly toxic.^3^ Therefore, novel and effective anticancer drugs with high safety effects are urgently required.

Epithelial‐to‐mesenchymal transition (EMT), which involves a switch in cellular phenotypic characteristics including the loss of cell polarity, the weakening of cellular adhesion and cytoskeleton remodelling, is one of the most important factors that contribute to the aggressive phenotype of SCLC.^4^ This process is characterized by an increase in E‐cadherin expression and reduction in vimentin expression.

EMT‐induced cells can acquire migratory properties that promote tumour progression and metastatic development. Although the process of EMT in different kinds of human malignant tumours has been described in previous studies,^5^, ^6^, ^7^ the molecular mechanism by which EMT is triggered in SCLC remains elusive. Previous studies reported that numerous signalling pathways are associated with the process of EMT in SCLC; these pathways include the Notch,^8^ Hedgehog^9^ and focal adhesion kinase (FAK) signalling pathways.^10^ Among these factors, the highly activated FAK signalling pathway is strongly believed to contribute to triggering EMT in SCLC.

FAK is a 125 kDa non‐receptor protein tyrosine kinase which is located at sites of integrin clustering in focal adhesions; therefore, FAK plays a central role in cellular processes that include migration and adhesion.^11^ FAK has many phosphorylated tyrosine sites, and these sites are key for the signal transduction function of FAK. The Y397 site in FAK is an autophosphorylation site that allows binding to src family kinases, which in turn phosphorylates other FAK residues, leading to downstream signalling. A large number of downstream signalling pathways, including the PI3K/Akt, RAF/JNK and ERK1/2 pathways, are mediated by FAK,^12^, ^13^, ^14^ However, few studies have focused on the underlying mechanism of EMT activation induced by the FAK signalling pathway in SCLC.

**Figure 1:**
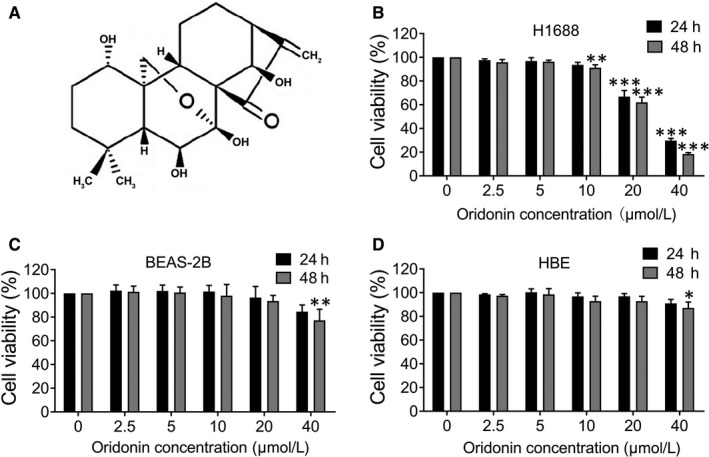
Effect of oridonin on the viability of H1688, BEAS‐2B and HBE cells. A, Chemical structure of oridonin. B, H1688, (C) BEAS‐2B and (D) HBE cells were treated with oridonin (0, 2.5, 5, 10, 20 and 40 μmol/L) for 24 and 48 h and assessed by MTT assay. The data represent the means ± SD of three independent experiments; *P < .05, **P < .01, *** *P* < .001 compared with the control

Natural products extracted from Chinese herb have recently received increasing attention in cancer therapy. Oridonin has a molecular formula of C_20_H_28_O_6_ (Figure 1A), an ent‐kaurane diterpenoid identified from the Chinese medicinal herb *Rabdosia rubescens.* It was reported that oridonin has multifunctional effects, including anti‐inflammatory, antibacterial and anticancer effects.^15^ In particular, the anticancer properties of oridonin have received a great deal of interest. The anticancer effects of oridonin include apoptosis induction, proliferation inhibition and cell migration via the regulation of multiple pathways, such as the Notch,^16^ hedgehog and integrin β1/FAK pathway.^17^ However, the effect of oridonin on cell migration in SCLC is unclear. Furthermore, the underlying mechanisms of oridonin on anticancer effects have not been clearly established.

Therefore, this study focused to investigate the role of oridonin on the migration of the H1688 cell and identified the molecular mechanism involved in this process.

## 2 MATERIALS AND METHODS

### 2.1 Chemicals and reagents

Oridonin was obtained from Aladdin (Shanghai Biochemical Technology Co., Ltd.). PF573228 (FAK inhibitor) and PD98059 (ERK1/2 inhibitor) were purchased from Selleck. Roswell Park Memorial Institute (RPMI) 1640 medium and foetal bovine serum (FBS) was obtained from Grand Island Biological Company. TRIzol reagent was obtained from Invitrogen. 3‐(4,5‐Dimethylthiazol‐2‐yl) ‐2,5‐diphenyltetrazolium bromide (MTT) and dimethyl sulphoxide (DMSO) were purchased from Sigma. Primary antibodies against the following proteins were used: E‐cadherin, vimentin, snail and slug, which were purchased from Abcam Co., Ltd.; FAK, p‐FAK^(Tyr397)^, ERK1/2 and p‐ERK1/2^(Thr202/Tyr204)^, which were obtained from Cell Signaling Technology Co., Ltd.; integrin β1 was purchased from ImmunoWay Biotechnology Company; and β‐actin and GAPDH were purchased from Santa Cruz Biotechnology Co., Ltd. Secondary anti‐mouse and anti‐rabbit antibodies were obtained from Pierce. Opti‐MEM serum‐free medium and lipofectamine RNAiMAX were obtained from Thermo Fisher Scientific.

### 2.2 Cell lines and cell culture

A human small cell lung cancer cell line (H1688), a human normal lung epithelial cell line (BEAS‐2B) and a human normal bronchial epithelial cell line (HBE) were obtained from Zhejiang University, School of Medicine. The three cell lines were cultured in RPMI 1640 complete medium contained with 10% FBS, 100 U/mL streptomycin and 100 U/mL penicillin. Cell was cultured at 37°C in a humidified atmosphere containing 5% CO_2_.

### 2.3 MTT assay for cell viability

The effect of oridonin on cell viability was assessed using the MTT assay. Briefly, cells (1 × 10^4^ cells/well) were plated in 96‐well culture plates. After being incubated overnight, the cells were treated with oridonin (0, 2.5, 5, 10, 20 and 40 μmol/L) for 24 and 48 hours. 10 μL MTT solution (5 mg/mL) was added to each well, and after 4 hours of incubation, the medium was discarded. DMSO (150 μL) was added to dissolve the resulting formazan crystals. The absorbance value at 570 nm was measured using an enzyme‐linked immunosorbent assay reader (Labsystems, Finland). The cell viability rate was calculated as follows: cell viability rate (%) = OD of test group/OD of control group × 100%.

### 2.4 Hoechst 33258 staining

Hoechst 33258 staining was used to detect apoptotic nuclei which were characterized by nuclear fragmentation. H1688 cells (1 × 10^5^ cells/well) were seeded onto 15‐mm microscope cover glasses in 6‐well culture plates. Then, the cells were treated with oridonin (0, 5, 10 and 20 μmol/L) for 24 hours. After being washed with PBS, the cells were fixed by using paraformaldehyde (4%) for 30 minutes, stained with Hoechst 33258 for 5 minutes in the dark and examined under a fluorescence microscope (Olympus).

### 2.5 Annexin and Propidium Iodide (PI) staining

H1688 cells were seeded in 6‐well culture plates. After 24 hours of oridonin treatment (0, 5, 10 and 20 μmol/L), the cells were harvested, and apoptosis levels were detected using Annexin V‐/PI staining kit (Yeasen Biotech Co., Ltd.). First, cells were collected and washed with PBS. Second, cells were resuspended 2 × 10^5^ cells/mL in 100 µL binding buffer and then incubated with 5 µL of Annexin V‐FITC and 10 µL of PI staining solution in the dark for 20 minutes at room temperature. Third, 400 µL of binding buffer was added. Samples were analysed using a Beckman CytoFLEX with CytExpert software and assessed according to the percentage of Annexin V‐PI‐positive cells.

### 2.6 Wound‐healing assay

H1688 cells were seeded in 6‐well culture plates using complete medium until the cell monolayer reached confluence. A scratch was made at the centre of each well using a 200‐μL pipette tip. The wounded monolayer was washed twice with PBS and further incubated with oridonin (0, 5 and 10 μmol/L), PF573228 (10 μmol/L) or PD98059 (10 μmol/L). The open wound was photographed under an inverted microscope at different time‐points (0 and 24 hours), and the width of the open wound in three fields was measured. The migration index was calculated as follows: migration index (%) = (‘0’ hour scratch width−‘24’ hours scratch width)/‘0’ hour scratch width × 100%.

### 2.7 Transwell migration assay

The ability of H1688 cells to migrate through polycarbonate filters (6.5 mm) with an 8 μm pore size was assessed according to a previous study with modifications.^18^ H1688 cells were suspended in serum‐free medium containing the indicated concentration of oridonin (0, 5 and 10 μmol/L), PF573228 (10 μmol/L) or PD98059 (10 μmol/L) in upper transwell chambers. The lower chambers were loaded with 10% FBS medium as a chemoattractant. After incubation for 24 hours, cells in the upper chamber were carefully removed, and cells which were in the lower chamber surface were fixed with 4% paraformaldehyde and stained with 0.5% crystal violet. Migrated cells were then photographed and counted in 3 random fields under a light microscope.

### 2.8 siRNAs

All siRNAs were purchased from GenePharma Co., Ltd. Control‐A scrambled siRNAs were used for negative control, respectively. All siRNAs consisted of scrambled sequences with no specific degradation of any cellular message. FAK, ERK1 and ERK2 siRNAs were used for specific gene expression knockdown. qRT‐PCR was used to assess the effect of siRNAs on suppressing target mRNA expression. The sequence of siRNA was used in this study is listed in Table 1.

**Table 1:** RNA oligo sequence

Gene	RNA oligo (5 ' to 3 ')
Negative control	F: 5′‐UUCUCCGAACGUGUCACGUTT‐3′
	R: 5′‐ACGUGACACGUUCGGAGAATT‐3′
FAK	F: 5′‐CCUAAGAGUUUACUGGAUUTT‐3′
	R: 5′‐AAUCCAGUAAACUCUUAGGTT‐3′
ERK1	F: 5′‐GACCGGAUGUUAACCUUUATT‐3′
	R: 5′‐UAAAGGUUAACAUCCGGUCTT‐3′
ERK2	F: 5′‐CCAUAUCUGGAGCAGUAUUTT‐3′
	R: 5′‐AAUACUGCUCCAGAUAUGGTT‐3′

### 2.9 RNA interference

H1688 cells were transfected with FAK siRNA, ERK1 siRNA, ERK2 siRNA or control non‐specific siRNA according to the manufacturer's instructions. Briefly, cells were plated in 6‐well culture plates. After being incubated overnight, siRNAs were diluted in Opti‐MEM serum‐free medium. Then, lipofectamine RNAiMAX was added to siRNA‐Opti‐MEM solution. After incubation for 10 minutes, siRNA‐lipid complex was added and incubated for 6 hours. Then, siRNA‐lipid complex was discarded and cells were cultured in RPMI 1640 complete medium for 48 hours. The expression of p‐FAK and p‐ERK1/2 was determined by Western blotting. The functional effects of siRNA on cell migration were evaluated by wound‐healing and transwell migration assay as described above.

### 2.10 Quantitative Real‐time PCR

TRIzol reagent was used to extract cell RNA, and the RNA concentration was measured spectrophotometrically. For reverse transcription, 1 μg RNA was added to the cDNA synthesis reaction system (20 μL) using cDNA Synthesis Kit (Takara). For qRT‐PCR analysis, the reaction mixture (20 μL) consisted of 2 μL cDNA, 10 μL of SYBR® Premix Ex Taq™ (Takara), 1 μL 2.5 U Taq DNA polymerase, 1 μL of 10 pmol/μL primer (Invitrogen) and 6 μL of ddH_2_O. The cDNA was denatured by heating to 94°C for 3 minutes. The template was amplified by 40 cycles of PCR of 95°C for 10 seconds, 60°C for 30 seconds and 72°C for 30 seconds, and a final extension at 72°C. The primers used in this study are listed in Table 2. For mRNA analysis, the relative level of target gene expression was determined using the cycle threshold (Ct) method and was normalized to the housekeeping gene used as internal control (GAPDH), using the 2−ΔCt method.

**Table 2:** Real‐time PCR primers

Gene	Primer sequences (5' to 3')
E‐cadherin	F: 5′‐GAGAACGCATTGCCACATACACTC‐3′
	R: 5′‐GGAAGAGCACCTTCCATGACAGAC‐3′
Vimentin	F: 5′‐TGAAGTGGATGCCCTTAAAGGAA‐3′
	R: 5′‐GCAGGCGGCCAATAGTGTCT‐3′
Snail	F: 5′‐CACCTCCAGACCCACTCAGATGT‐3′
	R: 5′‐GCAGGGACATTCGGGAGAAGGT‐3′
Slug	F: 5′‐GCGAACTGGACACACATACAGTG‐3′
	R: 5′‐GCTGAGGATCTCTGGTTGTGGT‐3′
FAK	F:5′‐CTCCTGGTGCAATGGAGCGAGTAT‐3′
	R: GCAGGTGACTGAGGCGGAATC‐3′
ERK1	F:5′‐CCCCCTAGCCCAGACAGACAT‐3′
	R:5′‐GGCTGGGCACAGTGTCCATT‐3′
ERK2	F:5′‐CTGTTCCCAAATGCTGACTCCAA‐3′
	R:5′‐CTCGTCACTCGGGTCGTAA‐3′
GAPDH	F: 5′‐CCATGACAACTTTGGTATCGTGGAA‐3′
	R: 5′‐GGCCATCACGCCACAGTTTC‐3′

### 2.11 Western blot analysis

Radio immunoprecipitation assay buffer (Beyotime Institute of Biotechnology) was used to extract the proteins in H1688 cells. Protein concentrations were evaluated by Bio‐Rad protein assay (Bio‐Rad Laboratories). 30 μg protein of each sample was transferred to PVDF membranes (Millipore Corporation). The membranes were blocked with 5% non‐fat milk in TBST buffer for 1 hour and incubated with primary antibodies (against E‐cadherin, vimentin, snail, slug, β‐actin, integrin β1, FAK, p‐FAK, ERK1/2 and p‐ERK1/2) at 4°C overnight. After three washes with TBST, the membranes were incubated with fluorescent secondary antibodies (LI‐COR Biotechnology, Lincoln) for 2 hours at room temperature. The signals of the bands were quantified using the VICTOR‐Z 1420 multilabel counter (EG&G Wallac). The results are expressed as the relative density. Equal loading protein in each lane was normalized to the loading β‐actin. All densitometry analyses performed in this study were conducted using ImageJ software (National Institute of Health).

### 2.12 Immunofluorescence assay

The localization of E‐cadherin and vimentin expression in the cells was observed by immunofluorescence staining. H1688 cells (1 × 10^5^ cells/well) were seeded onto 15‐mm coverslips in 6‐well culture plates. Then, the cells were incubated with oridonin (0, 5 and 10 μmol/L) for 24 hours. After this, the cells were harvested and washed twice with PBS, fixed with paraformaldehyde (4%) for 30 minutes and permeabilized with 0.2% Triton X‐100 for 10 minutes at room temperature. Cells were blocked with BSA for 30 minutes and incubated with primary antibodies against E‐cadherin and vimentin overnight at 4°C. After washing with PBS, the cells were incubated with FITC or TRITC conjugated secondary antibody for 1 hour. Finally, nuclei were labelled with DAPI for 10 minutes. Pictures were acquired using fluorescence microscopy (Olympus).

### 2.13 Animals and tumour xenograft model

Fifteen male BALB/c nude mice (18‐20 g, aged 5 weeks) were provided by the Zhejiang Chinese Medical University Laboratory Animal Research Center. All procedures in this animal study were granted by the Ethics Committee of Hangzhou Medical College. Mice were housed in standard conditions: 21 ± 2°C and 45 ± 10%, a 12‐hours light/12‐hours dark cycle with standard food and water ad libitum. H1688 cells (1 × 10^7^) in 200 μL of PBS were injected into the backs of the mice under the skin. When most tumour volumes reached approximately 200 mm^3^ (approximately one week after injection), the mice were randomly divided into three groups: a control group and oridonin groups with a low dosage (5 mg/kg/d) and a high dosage (10 mg/kg/d). Oridonin was dissolved in DMSO at the 5 or 10 mg/kg, then diluted with saline to 0.5 or 1 mg/kg, and the oridonin groups received an intraperitoneal injection of 5 or 10 mg/kg oridonin every other day for 21 days. The control group was administered the same volume of saline. The mouse weights and tumour volumes were measured every other day. Tumour volumes were calculated using the following formula: (length × width^2^)/2.

### 2.14 Haematoxylin and eosin (HE) staining

After 21 days of injection, the mice were anaesthetized using a 1% pentobarbital sodium salt solution (30 mg/kg, Sigma‐Aldrich). Blood samples were collected for biochemical serum analysis. The harvested tumours were weighed and fixed in a 4% buffered paraformaldehyde solution for 24 hours. Fixed tissues were embedded in paraffin and cut into 4‐μm sections. The sections were subjected to haematoxylin and eosin staining according to a standard protocol. Major organs (the lung, liver, heart and kidney) were collected, fixed in a 4% buffered paraformaldehyde solution and paraffin‐embedded for HE staining. The results were visualized and photographed with a light microscope.

### 2.15 Immunohistochemistry analysis

The expression levels of E‐cadherin, vimentin and p‐FAK^(Tyr397)^ were detected by immunohistochemical staining in xenograft tumours. Briefly, 4‐μm tissue sections were dried overnight at 37°C, deparaffinized in xylene, rehydrated with gradient alcohol and immersed in 10 mmol/L citric acid (pH 6.0). The sections were incubated with primary antibodies against E‐cadherin, vimentin and p‐FAK ^(Tyr397)^ overnight at 4°C. Then, the sections were incubated in biotinylated second antibody (1:200, Boster Biological Technology) for 2 hours at room temperature. After washing in PBS, these sections were incubated in an avidin‐biotin‐peroxidase complex solution (ABC, 1:100, Boster Biological Technology). The results were visualized with diaminobenzidine and photographed with a light microscope.

### 2.16 Statistical analysis

All data are shown as the mean ± standard deviation (SD). The analyses were performed using GraphPad Prism. Significant difference was determined by one‐way ANOVA. The intergroup differences were determined by Tukey's post hoc test for multiple comparisons. A *P* value of <.05 was regarded as statistically significant.

## 3 RESULTS

### 3.1 The cell viability was reduced by high concentrations of oridonin in H1688 cells but not in normal cells

The cytotoxic effect of oridonin on cells was determined by MTT assay. As shown in Figure 1B, treatment with lower concentrations of oridonin (0, 2.5, 5 and 10 μmol/L) for 24 hours did not affect the cell viability of H1688 cells; however, high concentrations of oridonin (20 and 40 μmol/L) significantly reduced cell viability for 24 and 48 hours (*P* < .05). Furthermore, we also examined the effect of oridonin on BEAS‐2B and HBE. In contrast, no marked cytotoxic effects were observed in these cell lines when they were exposed to the same concentrations of oridonin (except for 40 μmol/L) for 24 and 48 hours (Figure 1C,D).

### 3.2 Apoptosis was not induced by the application of lower concentrations of oridonin

**Figure 2:**
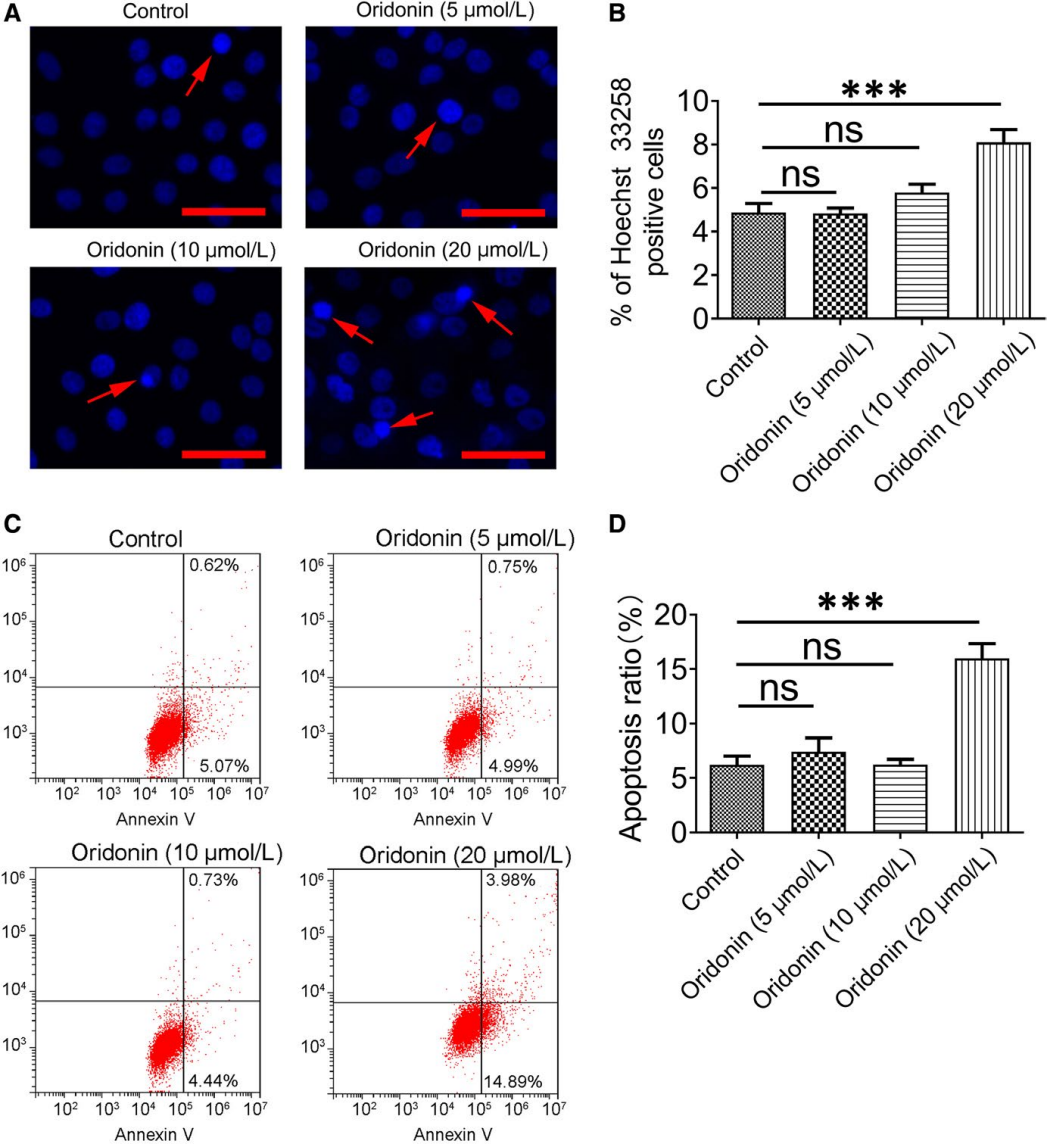
The effect of oridonin on the apoptosis of H1688 cells was determined with Hoechst 33258 and Annexin V‐TITC/PI staining. A, H1688 cells were treated with oridonin (0, 5, 10 and 20 μmol/L) for 24 h, and morphology changes in cell nuclei were observed by Hoechst 33258 staining. A marked increase in apoptotic cells exhibiting a heterogeneous intensity, chromatin condensation and fragmentation appeared after 24‐h treatment with 20 μmol/L oridonin. Scale bars = 50 μm. The pooled data from nine sections for each group are summarized in (B). C, Annexin V‐TITC/PI staining measured by flow cytometry revealed a significant increase in the apoptotic cell ratio in the 20 μmol/L oridonin treatment group. D, Quantification of the results of flow cytometry. ***P < .001; ns, not significant

As a high concentration of oridonin was reported to induce apoptosis,^19^ therefore, to investigate whether lower concentrations of oridonin could induce apoptosis in H1688 cells, some experiments were performed. First, as demonstrated in Figure 2A, the result of Hoechst staining showed that the percentage of apoptotic cells characterized by nuclear condensation and fragmentation was approximately 4.88 ± 1.22% in the control group (Figure 2B). Meanwhile, the percentage of apoptotic cells in the oridonin treatment groups (5 and 10 μmol/L) was 4.83 ± 0.74% and 5.80 ± 1.11%, respectively, and there was no difference in the percentage of apoptotic cells between these three groups. However, the percentage of apoptotic cells in the group treated with 20 μmol/L oridonin was 8.10 ± 1.79%, which was significantly higher than that in the control group (*P* < .01, Figure 2C,D). Second, there was only a small percentage of early apoptotic cells in the control and oridonin treatment groups (5 and 10 μmol/L); however, the apoptotic ratio in the 20 μmol/L oridonin treatment group was apparently increased compared with the control group according to flow cytometry (*P* < .01, Figure 2,D).

The above results showed that lower concentrations of oridonin (5 and 10 μmol/L) were not cytotoxic and had no effect on apoptosis induction. Therefore, non‐cytotoxic concentrations of oridonin (5 and 10 μmol/L) were used in subsequent experiments.

### 3.3 Oridonin inhibited migration and reduced EMT in H1688 cells

**Figure 3:**
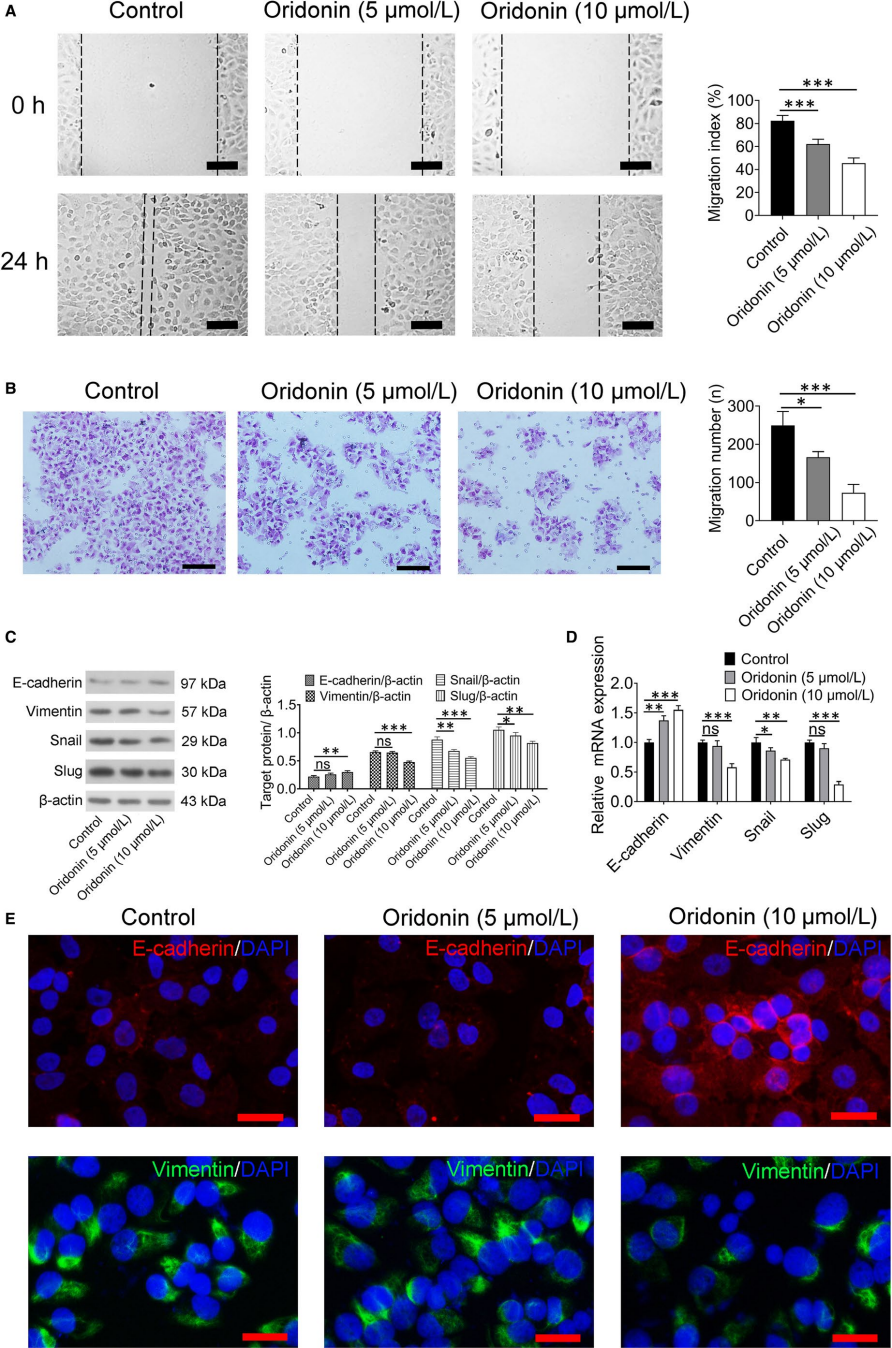
The migration of H1688 cells was reduced by oridonin treatment via suppression of the EMT process. A, Cell migration in H1688 cells treated with oridonin (5 and 10 μmol/L) and control H1688 cells was determined by wound‐healing assay. Representative sections indicating a significant decrease in migration index after 24 h of oridonin treatment (left); scale bars = 100 μm. Analysis of data representing three independent experiments (right). B, Cell migration was determined by transwell assay after oridonin (0, 5 and 10 μmol/L) treatment for 24 h. Cells that travelled through the transwell membrane in each group were stained with crystal violet and obviously decreased after 24 h of oridonin (5 and 10 μmol/L) treatment (left); scale bars = 100 μm. Analysis of data representing three independent experiments (right). C, Western blotting was applied and showed that the protein expression of E‐cadherin was significantly increased after 24 h of oridonin (5 and 10 μmol/L) treatment, whereas the expression of vimentin, snail and slug was decreased in the oridonin‐treated groups. D, Relative mRNA expression of E‐cadherin, vimentin, snail and slug in H1688 cells was analysed by quantitative real‐time PCR and normalized to GAPDH mRNA expression. The data represent three independent experiments. E, Immunofluorescent staining was performed and showed that the expression of E‐cadherin (red) was increased and the expression of vimentin (green) was decreased in H1688 cells treated with oridonin (10 μmol/L) for 24 h. * *P* < .05, ** *P* < .01, *** *P* < .001

Migration is one of the important characteristics of EMT. As shown in Figure 3A, the wound‐healing assay showed that treatment with oridonin (5 and 10 μmol/L) for 24 hours resulted in a reduction in cell migration because migration index was significantly reduced than that in the control group (*P* < .05). Furthermore, as shown in Figure 3B, the number of migrated cells was markedly reduced after oridonin treatment (5 and 10 μmol/L) for 24 hours. It indicated that oridonin can inhibit H1688 cancer cell migration.

As some proteins, including E‐cadherin, vimentin, snail and slug, are involved in triggering the EMT process, the expression of these proteins was measured. As shown in Figure 3C,D, the expression levels of vimentin, snail and slug, as determined by real‐time PCR and Western blotting, were decreased in cells treated with high concentrations of oridonin, whereas E‐cadherin expression was increased in high dosage‐oridonin treatment groups (10 μmol/L) when compared with the control group (*P* < .001). Furthermore, there are an increase in E‐cadherin expression and a decrease in vimentin expression after oridonin treatment (10 μmol/L), as shown by immunofluorescence staining, which was consistent with the results of Western blotting (Figure 3E).

### 3.4 Oridonin inhibited the FAK‐ERK1/2 signalling pathway in H1688 cells

**Figure 4:**
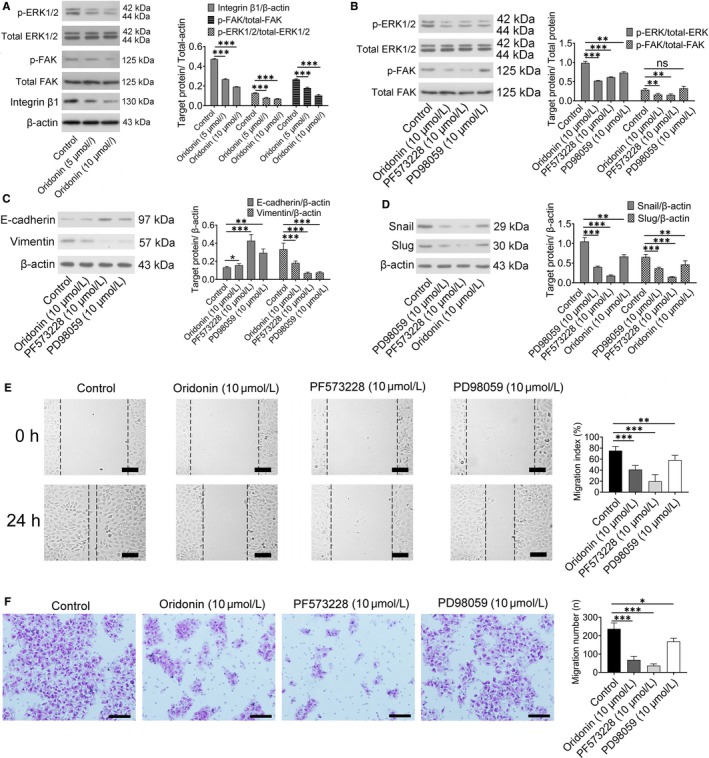
The anti‐EMT effect of oridonin was mediated by inactivation of the FAK/ERK1/2 signalling pathway. A, Representative Western blots showing that integrin β1 p‐FAK and p‐ERK1/2 levels were reduced in the oridonin (5 and 10 μmol/L) treatment groups (left). Bar graphs represent quantitative differences in the expression of integrin β1 p‐FAK and p‐ERK1/2 (right). Data represent the means ± SD of four independent experiments. B, Representative Western blots showing that PF573228 (10 μmol/L), an inhibitor of FAK, reduced the expression of p‐FAK and p‐ERK1/2, which was similar to the effect of oridonin (10 μmol/L). In addition, the expression of p‐ERK1/2 was down‐regulated by PD98059 (left), and data represent the means ± SD of four independent experiments (right). C, The protein expression of E‐cadherin and vimentin was detected by Western blotting (left), and the data represent the means ± SD of four independent experiments (right). D, The protein expression of snail and slug was detected by western blotting, and the data represent the means ± SD of four independent experiments (right). E, Representative sections indicated that the migration index wound space was significantly decreased after 24 h of oridonin (10 μmol/L), PF573228 (10 μmol/L) or PD98059 (10 μmol/L) treatment (left); scale bars = 100 μm. Analysis of data representing three independent experiments (right). F, Cells that travelled through the transwell membrane in each group were stained with crystal violet and obviously decreased after 24 h of oridonin (10 μmol/L), PF573228 (10 μmol/L) and PD98059 (10 μmol/L) treatment (left); scale bars = 100 μm. Analysis of data representing three independent experiments (right). *P < .05; **P < .01; ***P < .001

To characterize the possible mechanism underlying the effect of oridonin on inhibiting H1688 cell migration, the expression of the FAK and ERK1/2 proteins, which are regulated by the FAK pathway, was examined. As shown by Western blotting and in Figure 4A, integrin β1, p‐FAK and p‐ERK1/2 expression was drastically decreased after exposure to oridonin (5, 10 μmol/L) for 24 hours compared with the control group (*P* < .001). To confirm that the protective effect of oridonin on cell migration indeed involves suppressing the FAK pathway, the FAK inhibitor PF573228 and the ERK1/2 inhibitor PD98059 were used. First, the expression of p‐FAK and p‐ERK1/2 was reduced by application of the FAK inhibitor PF573228; however, the ERK1/2 inhibitor PD98059 could only down‐regulate the expression of p‐ERK1/2 (Figure 4B) as ERK1/2 is a downstream protein of FAK. Next, Western blotting showed that the expression of E‐cadherin was obviously increased after PF573228 and PD98059 treatment, whereas the expression levels of vimentin, snail and slug were significantly decreased when compared with the control group (*P* < .001, Figure 4C,D), which was similar to the effect of oridonin. Finally, migration index was highest in the control group (*P* < .001, Figure 4E), whereas the number of migrated cells in the control group was greater than that in the other groups (*P* < .001, Figure 4F). Although migration index and the number of migrated cells were reduced by the administration of oridonin, PF573228 and PD98059, the effect of PD98059 against EMT was weaker than that of PF573228, which suggests that many downstream signalling pathways besides the ERK1/2 pathway are involved after FAK activation.

### 3.5 The effect of siRNA‐mediated knockdown on cell migration

**Figure 5:**
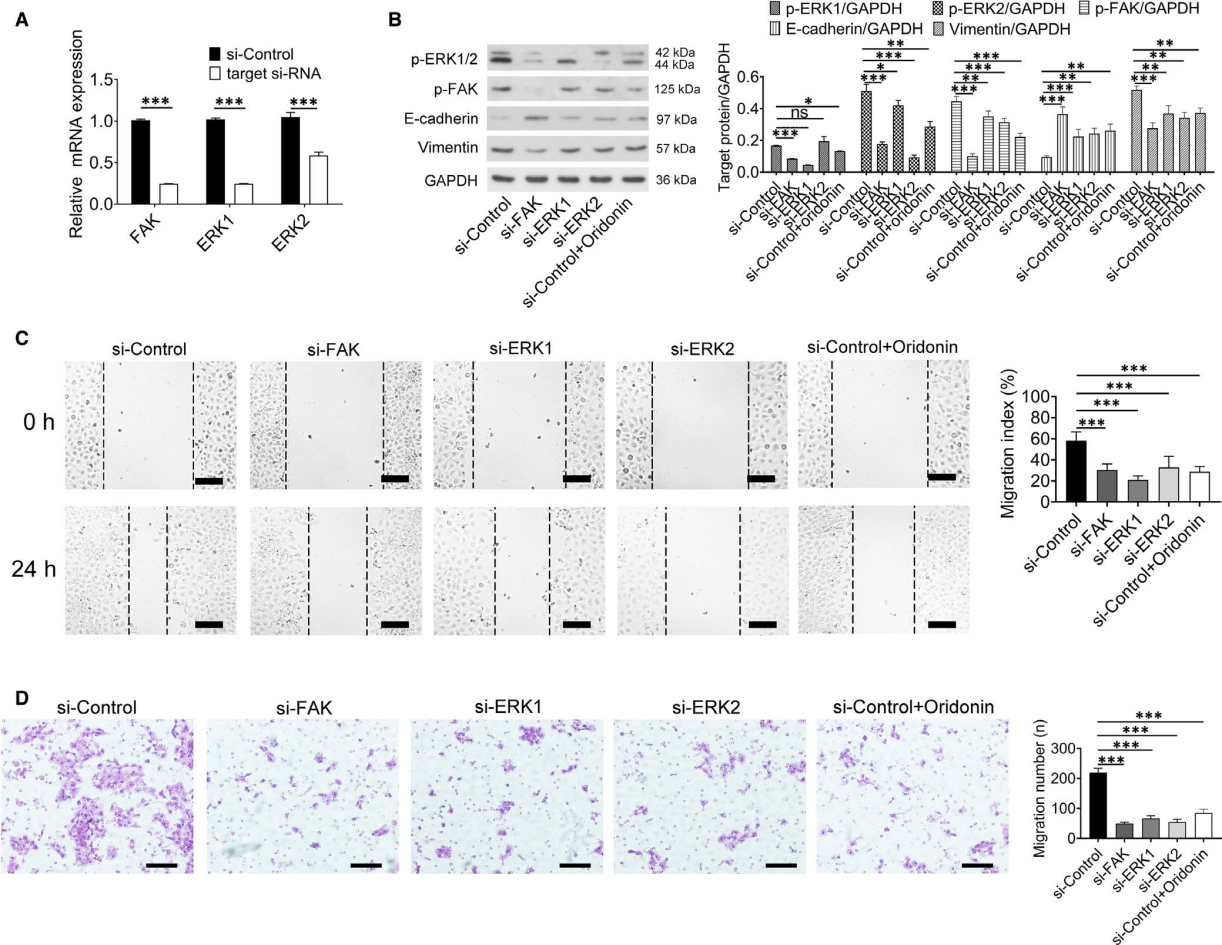
Specific knockdown of FAK, ERK1 and ERK2 could reduce the migration of H1688 cells. A, The mRNA levels of FAK, ERK1 and ERK2 were significantly reduced after 48 h of siRNA‐mediated knockdown. B, The protein expression of p‐ERK1, p‐ERK2, p‐FAK, E‐cadherin and vimentin was detected by Western blotting (left), and the data represent the means ± SD of four independent experiments (right). C, Representative sections indicated that the migration index wound space was significantly decreased after siRNA‐mediated knockdown (left); scale bars = 100 μm. Analysis of data representing three independent experiments (right). D, Cells that travelled through the transwell membrane in each group were stained with crystal violet and obviously decreased after siRNA‐mediated knockdown (left); scale bars = 100 μm. Analysis of data representing three independent experiments (right). *P < .05; **P < .01; ***P < .001

In order to confirm the role of FAK‐ERK1/2 signalling pathway on cell migration, RNA interference was used to suppress the expression of FAK and ERK1/2. From the result of Figure 5A, siRNA treatment caused significant down‐regulation of target gene in 48 hours. As shown in Figure 5B, the expression of p‐FAK and p‐ERK1/2 was all reduced after incubating si‐FAK; meanwhile, ERK1 and ERK2 siRNA treatment caused significant down‐regulation of p‐ERK1 and p‐ERK2 expression, respectively. Furthermore, the expression of E‐cadherin was obviously increased and the expression of vimentin was significantly reduced after FAK and ERK1/2 siRNA treatment (Figure 5B). Finally, migration index and the number of migrated cells were reduced in the FAK and ERK1/2 siRNA group when compared with control group (Figure 5C,D). The inhibition effect of cell migration was even stronger than that in oridonin group.

Taken together, these results provide evidence that EMT activity is inhibited by oridonin via blockade of the FAK‐ERK1/2 signalling pathway.

### 3.6 Oridonin reduced the tumour volume in vivo via up‐regulating the expression of E‐cadherin and down‐regulating the expression of vimentin

**Figure 6:**
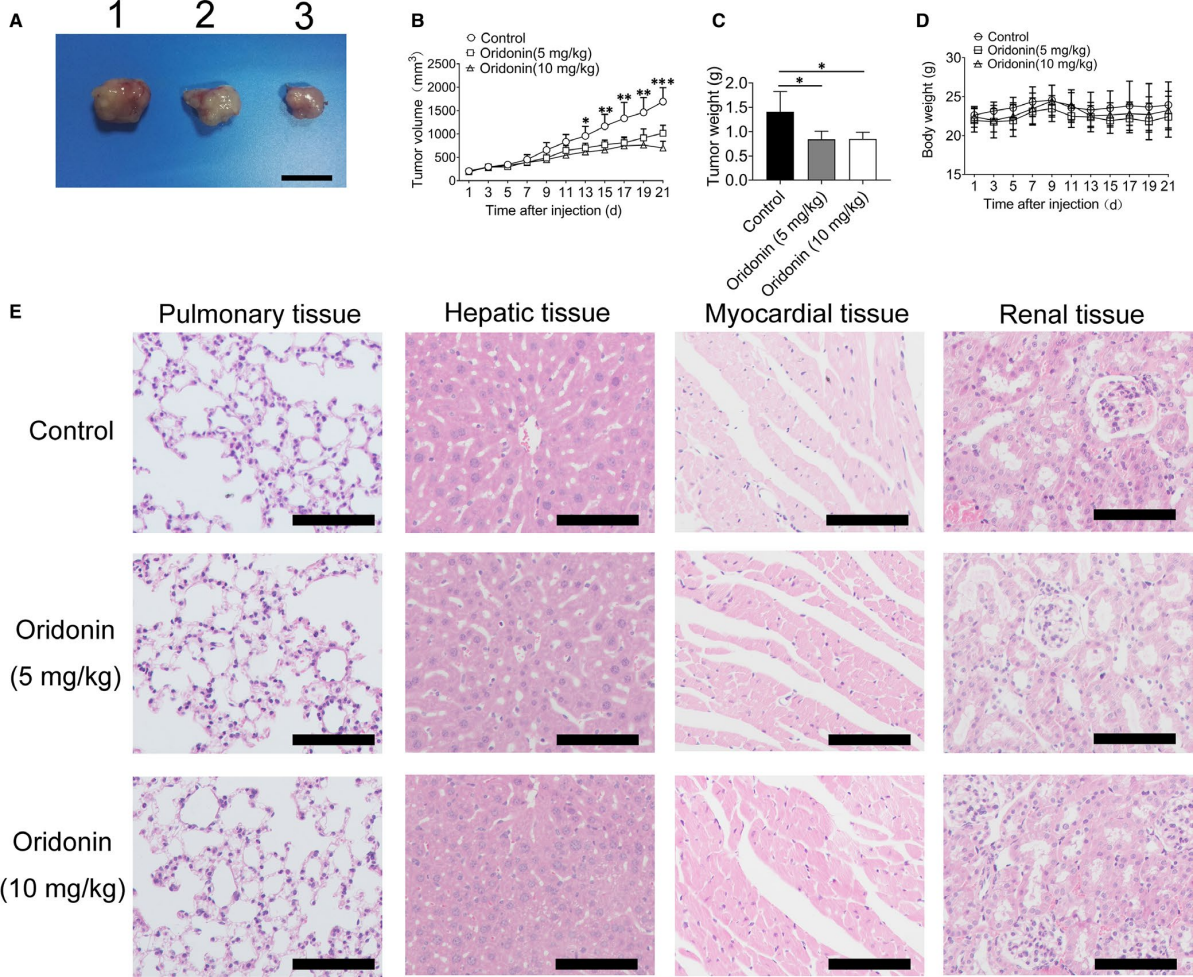
The daily injection of oridonin could reduce tumour weight and volume and exhibited a high level of safety. A, Representative images of tumour from three groups. 1: control group, 2: oridonin (5 mg/kg) group and 3: oridonin (10 mg/kg) group. Scale bars = 1 cm. B, The tumour width (W) and length (L) were measured by a micrometre every other day. Tumour volumes were calculated as (L × W^2^)/2. C, The tumour weight was significantly reduced after treatment with oridonin (5 and 10 mg/kg). D, Body weight was not obviously altered after treatment with oridonin (5 and 10 mg/kg). E, The administration of oridonin (5 and 10 mg/kg) had no effect on the health of nude mice, as assessed by body weight. F, H&E staining to examine major organs in oridonin‐treated mice. There was no obvious pathological change in the lung, heart and kidney; however, the hepatic cord was narrowed following treatment with a high concentration of oridonin; scale bars = 100 μm. *P < .05; **P < .01; ***P < .001

To verify the antitumor effects of oridonin in vivo, nude mice (BALB/c‐nu/nu) bearing H1688 tumour xenografts were treated with placebo or oridonin. As shown in Figure 6A‐B, the tumour volume in mice treated with oridonin at doses of 5 and 10 mg/kg was apparently reduced compared with that in the control group after injection for 13 days (*P* < .01). Meanwhile, the tumour weight was also decreased in the oridonin‐treated groups after injection for 21 days (Figure 6C). It indicated that oridonin also inhibits the growth of small lung cancer cells in *vivo*; meanwhile, the toxicity of oridonin was also assessed by a set of several experiments. First, body weight was not obvious loss after oridonin treatment (Figure 6D). Second, the morphologies of the lung, heart and kidney did not significantly differ between the control and oridonin‐treated groups; however, the hepatic cord was narrowed following treatment with a high concentration of oridonin (10 mg/kg, Figure 6E). Finally, some biochemical serum parameters were significantly different between these groups (Table 3). The alanine aminotransferase (ALP) level was significantly increased after oridonin (5 and 10 mg/kg) treatment, which suggested hepatotoxicity. In addition, the lactate dehydrogenase (LDH) level was significantly decreased in the oridonin (10 mg/kg) group. Taken together, the results of our study demonstrated that oridonin at a dose of 5 mg/kg can inhibit tumour growth with relative safety.

**Table 3:** Effects of oridonin treatment on the serum levels of biochemical parameters in the tumour xenograft model (n = 5)

*Groups*	ALT (IU/L)	AST (IU/L)	ALP (IU/L)	BUN (mmol/L)	CREA (μmol/L)	UA (μmol/L)	CK (IU/L)	LDH (IU/L)
Control	18.12 ± 1.26	94.10 ± 9.50	76.63 ± 20.03	10.91 ± 2.56	42.39 ± 2.09	219.61 ± 19.53	461.89 ± 158.14	1927.57 ± 332.90
Oridonin (5 mg/kg)	36.36 ± 9.28*	133.72 ± 8.94	92.56 ± 5.81	13.39 ± 1.05	45.29 ± 2.74	346.05 ± 36.10	394.46 ± 157.63	1680.71 ± 248.64
Oridonin (10 mg/kg)	38.88 ± 13.9*	144.30 ± 52.52	79.18 ± 18.19	12.33 ± 0.44	43.84 ± 1.22	396.85 ± 36.11	358.10 ± 89.67	1241.06 ± 261.68*

Note

Data are presented as the mean ± SD (n = 5).

Abbreviations: ALP, alkaline phosphatase; ALT, alanine aminotransferase; AST, aspartate aminotransferase; BUN, blood urea nitrogen; CK, creatinine kinase; CREA, creatinine; LDH, lactate dehydrogenase; UA, uric acid.

* *P* < .05 vs control group.

** *P* < .01 vs control group.

**Figure 7:**
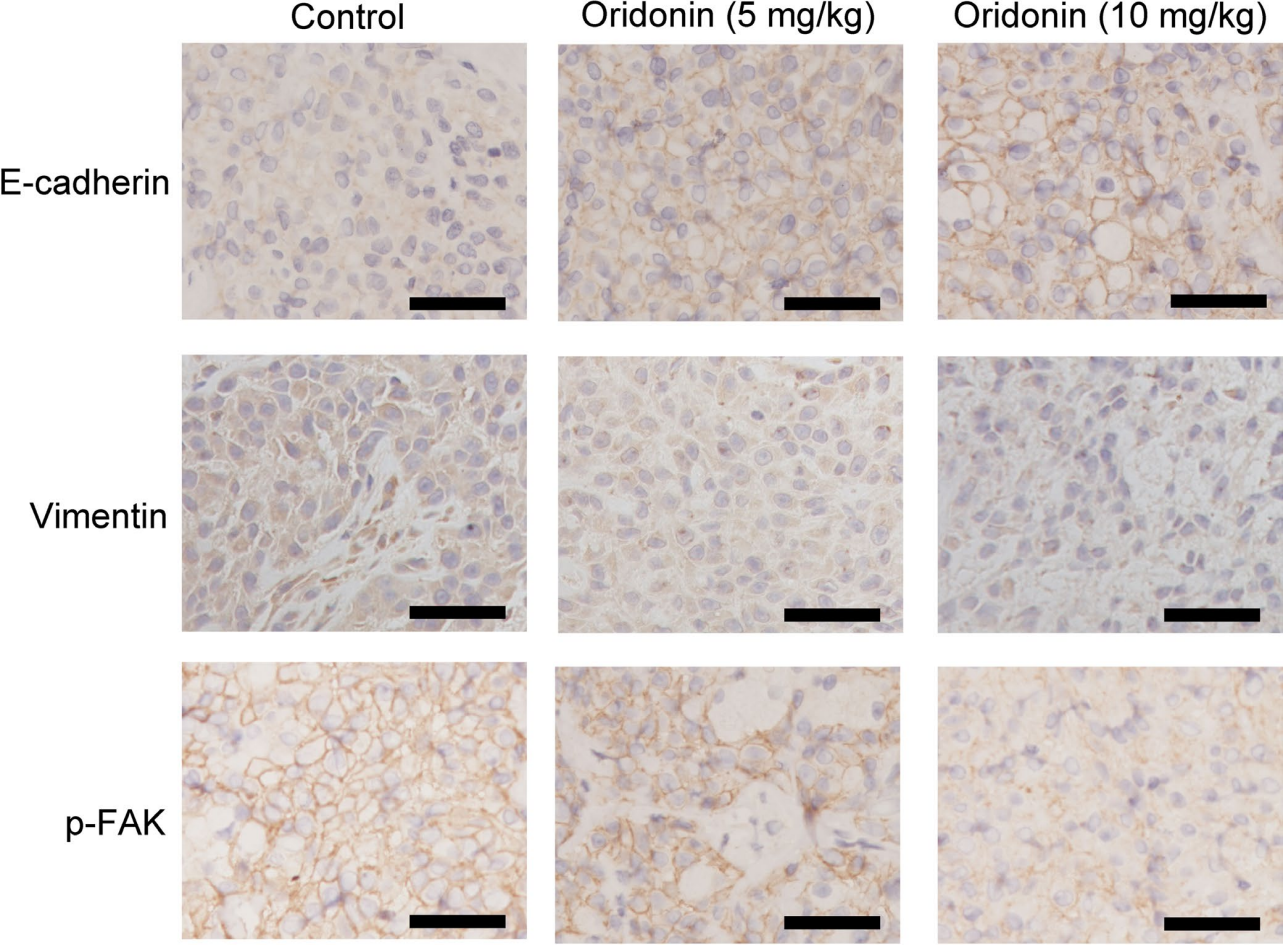
Histochemical analysis was used to detect the effect of oridonin on the expression of E‐cadherin, vimentin and p‐FAK in vivo in a nude mouse xenograft model. Scale bars = 50 μm

Additional histochemical analysis of the tumours in the three groups showed that oridonin‐treated tumours had higher levels of E‐cadherin than control H1688 cell tumours (Figure 7). Furthermore, the expression levels of both vimentin and p‐FAK^(Tyr397)^ were low and decreased in the oridonin groups. These data suggested that oridonin treatment reduces EMT properties in vivo.

## 4 DISCUSSION

Although a number of studies have demonstrated the antiproliferative effects of oridonin in a variety of cancer cells,^20^, ^21^ few studies have investigated the effect of oridonin on H1688, an SCLC cell line. The present study clearly demonstrates that oridonin can inhibit H1688 cell proliferation; furthermore, the intraperitoneal administration of oridonin at a dosage of 5 mg/kg caused a significant decline in tumour volume in lung cancer xenograft‐bearing nude mice associated with suppression of the FAK signalling pathway.

Enhanced cell migration is an important process for the metastasis of cancer cells and also a critical enhancing factor resulting in high mortality.^22^ As shown by some recent studies, some traditional Chinese medicines have been identified as effective anticancer drugs for various cancers 23, 24; however, the specific protective mechanisms of different herbal medicines are not the same. *R rubescens* has been used in traditional Chinese medicine for oesophageal cancer treatment in China for a long time.^25^ Oridonin, one of the most important active ingredients of *R rubescens*, was revealed to have a potential therapeutic effect against the migration of four selected cancer cell lines (MGC‐803, MCF‐7, PC‐3 and Eca‐109 cells) 20; however, the effect of oridonin on the H1688 SCLC cell line had not been investigated.

As discussed above, SCLC cells are characterized by high rates of migration; therefore, the effects of oridonin on the migration of the H1688 SCLC cell line were first investigated. In the present study, cell viability was reduced, and cell apoptosis was induced after the administration of 20 or 40 μmol/L oridonin for 24 hours, which suggested obvious cytotoxicity in H1688 lung cancer cells under treatment with high concentrations of oridonin. However, it was interesting that these concentrations of oridonin did not affect normal human bronchial epithelial cells, which is consistent with the results of a previous study showing the differential cytotoxic effects of oridonin on malignant and normal cells.^26^ Therefore, to elevate the safety of oridonin treatment, lower concentrations of oridonin (5 and 10 μmol/L) were assessed, which demonstrated that neither cell viability nor apoptosis was significantly altered under treatment with oridonin at these two lower concentrations.

Cell migration is one of the most important characteristics for aggressive cancer.^27^ In the present study, oridonin (5 and 10 μmol/L) significantly inhibited H1688 cell migration. First, wound‐healing assays demonstrated that cell migration index was significantly reduced in the oridonin treatment group. Second, it showed a reduction in the number of migrated cells in the oridonin treatment groups by using transwell migration assays, which demonstrated the inhibitory effect of oridonin on the migration of H1688 cells.

EMT is an essential step associated with cancer migration as it could enhance the ability of cancer cells to migrate.^28^ Therefore, we have been suggested that the inhibitory effect of oridonin on cell migration is associated with EMT. One of the hallmarks of EMT is the functional loss of E‐cadherin, which is associated with epithelial‐to‐mesenchymal transition in cancer.^29^ A previous study demonstrated that reduced E‐cadherin decreases the strength of cellular adhesion, resulting in increased cellular motility^30^; however, the overexpression of E‐cadherin was sufficient to prevent migration. Therefore, E‐cadherin plays a critical role in migratory ability. In our study, the expression of E‐cadherin was reduced in SCLC; however, the expression of E‐cadherin was increased after the application of oridonin. As previous studies have found that cell migration and EMT were inhibited by oridonin in pancreatic cancer^31^ and breast cancer,^32^ we have been suggested that the effect of oridonin on inhibiting EMT is associated with increased E‐cadherin expression.

To investigate the mechanism by which oridonin increased the expression of E‐cadherin in this study, the transcription factors snail and slug were investigated as these two proteins bind to the E‐cadherin promoter to suppress E‐cadherin expression.^33^ The results showed that the expression of snail and slug was significantly decreased by oridonin when compared with their expression in the control group.^30^ Furthermore, the expression of vimentin was significantly decreased after the application of oridonin. Vimentin is an intermediate filament (IF) protein that promotes the migration of different cell types.^34^ Taken together, it demonstrated that oridonin affects EMT in H1688 cells via modulating the expression of E‐cadherin and vimentin. These results indicated that the inhibition of EMT is involved in the antimigratory effect of oridonin.

A recent study demonstrated that oridonin inhibits MDA‐MB‐231 human breast cancer cell migration and invasion in vitro possibly by decreasing the levels of local adhesion kinase (FAK).^17^ FAK, a non‐receptor protein tyrosine kinase, is a key component in initiating downstream signalling that promotes cell migration.^35^ According to a previous study, both FAK and phospho‐FAK are highly expressed in SCLC cell lines,^10^ and the increased expression of phospho‐FAK was correlated with spreading, adhesion and migration in SCLC cells. In this study, the expression of phospho‐FAK was decreased in the oridonin group, which suggested that the effect of oridonin on inhibiting cell migration is also associated with the FAK signalling pathway. Although the specific mechanism of oridonin on inactivating FAK phosphorylation is not fully classified, one previous literature has demonstrated that oridonin inhibit FAK though reducing the expression of integrin β1 which was upstream of FAK.^17^ In this study, the expression of integrin β1 was indeed reduced after oridonin application.

As phospho‐FAK directly activates downstream effectors such as ERK1/2, a set of experiments were performed to delineate whether FAK‐ERK1/2 signalling plays a significant role in the migration and EMT status of H1688 cells. First, the expression of phospho‐ERK1/2 was examined after the administration of oridonin. The total levels of FAK and ERK1/2 were not affected, but the levels of phospho‐FAK and ERK1/2 were reduced by oridonin. According to previous study, NF‐κB p65, one target‐activated protein of ERK1/2, could bind on slug and snail promoter regions to increase these two gene expressions.^36^ Therefore, we have been suggested that the underlying mechanism of reducing the expression of snail and slug by oridonin was mediated by inhibiting ERK1/2‐ NF‐κB.

Second, two inhibitors, PF573228 and PD98059, were used in the present study. PF573228, an inhibitor of FAK, could indeed decrease the phosphorylation of FAK and ERK1/2; meanwhile, cell migration and EMT were also suppressed in cells pre‐treated with PF573228, and a similar phenomenon was observed in the group treated with PD98059 (an inhibitor of ERK1/2), except for the decreased phosphorylation of FAK. All the evidence shows that the anti‐EMT activity of oridonin is mediated through modulation of the FAK‐ERK1/2 signalling pathway. However, that the effect of PD98059 on inhibiting cell migration was decreased is interesting. This result suggests not only that the ERK1/2 signalling pathway targets a downstream protein of FAK but also that although the FAK‐ERK1/2 signalling pathway plays a critical role in inhibiting H1688 cell migration, another signalling pathway is involved.

Finally, FAK and ERK1/2 siRNA were used to suppress FAK‐ERK1/2 signalling pathway. The results showed that siRNA treatment had the effect on inhibiting cell migration. Therefore, the effect of oridonin on inhibition of EMT was indeed associated with suppressing FAK‐ERK1/2 signalling pathway.

Animal experiments showed that oridonin can clearly reduce the tumour volume and weight after oridonin treatment; however, oridonin, especially at high concentrations, had obvious cytotoxic effects on hepatic tissue and myocardial tissue based on morphological observations and the results of serum biochemical analysis. This finding suggests prudence in selecting the dose of oridonin for clinical use.

In summary, our data demonstrated that oridonin can significantly inhibit the migration and EMT of SCLC cells both in vivo and in vitro. These inhibitory effects are at least partially mediated by suppression of the FAK‐ERK1/2 signalling pathway. Thus, oridonin is a potentially useful antimetastatic agent for future SCLC treatment.

## CONFLICT OF INTEREST

The authors declare that they have no conflict of interest.

## AUTHOR CONTRIBUTIONS

LHX and JC designed the study. YZX collected and analysed the data. WJX and SSZ performed the histological staining and immunofluorescence staining. WJX contributed wound‐healing assay and transwell migration assay. The tumour xenograft model was performed by ZCZ. LHX, YLB and JC performed the experiments, including Western blot, real‐time PCR and flow cytometry. LHX drafted and wrote the manuscript. JC revised the manuscript. All authors read and approved the final manuscript.

## Data Availability

The data sets generated and analysed during this study are available from the corresponding author (Jian Chen) on reasonable request.
